# Maternal CXCR4 deletion results in placental defects and pregnancy loss mediated by immune dysregulation

**DOI:** 10.1172/jci.insight.172216

**Published:** 2023-11-08

**Authors:** Fang Lyu, Chase Burzynski, Yuan yuan Fang, Aya Tal, Alice Y. Chen, Jacqueline Kisa, Kriti Agrawal, Yuval Kluger, Hugh S. Taylor, Reshef Tal

**Affiliations:** 1 Department of Obstetrics, Gynecology and Reproductive Sciences, and; 2 Department of Pathology, Yale University School of Medicine, New Haven, Connecticut, USA.; 3 Program of Applied Mathematics, Yale University, New Haven, Connecticut, USA.

**Keywords:** Reproductive Biology, Bone marrow, Chemokines, Obstetrics/gynecology

## Abstract

CXCR4 is a key regulator of the development of NK cells and DCs, both of which play an important role in early placental development and immune tolerance at the maternal-fetal interface. However, the role of CXCR4 in pregnancy is not well understood. Our study demonstrates that adult-induced global genetic CXCR4 deletion, but not uterine-specific CXCR4 deletion, was associated with increased pregnancy resorptions and decreased litter size. CXCR4-deficient mice had decreased NK cells and increased granulocytes in the decidua, along with increased leukocyte numbers in peripheral blood. We found that CXCR4-deficient mice had abnormal decidual NK cell aggregates and NK cell infiltration into trophoblast areas beyond the giant cell layer. This was associated with low NK cell expression of granzyme B, a NK cell granule effector, indicative of NK cell dysfunction. Pregnancy failure in these mice was associated with abnormalities in placental vascular development and increased placental expression of inflammatory genes. Importantly, adoptive BM transfer of WT CXCR4^+^ BM cells into CXCR4-deficient mice rescued the reproductive deficits by normalizing NK cell function and mediating normal placental vascular development. Collectively, our study found an important role for maternal CXCR4 expression in immune cell function, placental development, and pregnancy maintenance.

## Graphical abstract

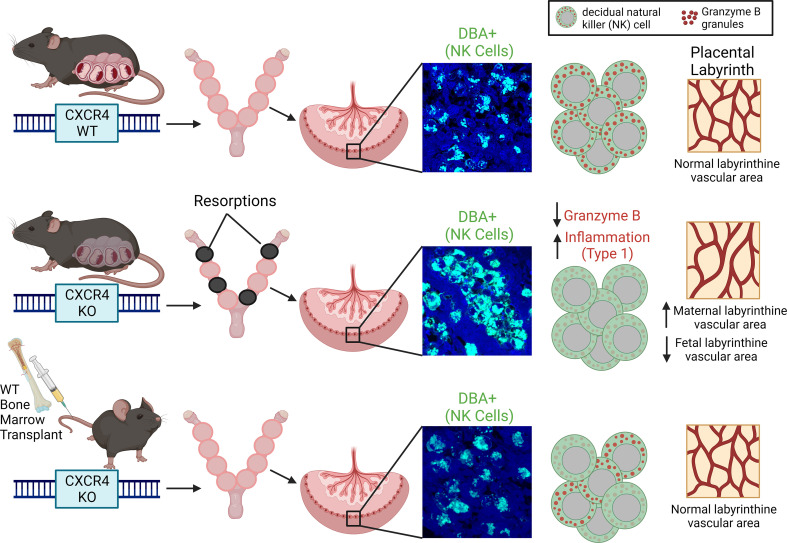

## Introduction

Pregnancy is an immunomodulatory process in which immune cells play an integral role, contributing to the communication between fetal trophoblast cells and maternal decidual cells leading to a successful pregnancy (1, 2). These immune cells communicate via production of cytokines, chemokines, and growth factors to establish a unique maternal-fetal immune environment that promotes fetal tolerance until parturition (3). Although there is increased understanding of the function of immune cells in reproduction, detailed mechanisms of how immune cell function is regulated within the maternal-fetal interface (MFI) are still not well understood.

NK cells account for ~70% of immune cells in the decidua during early pregnancy (4). These NK cells participate in trophoblast invasion and spiral artery remodeling, which are crucial for successful uterine decidualization, embryo implantation, and placentation (4–7). Macrophages account for 20%–30% of decidual leukocytes in the first trimester, playing an important role in the inhibition of inflammation and promoting maternal-fetal immune tolerance (8). T cells, especially CD4^+^ Tregs, enhance trophoblast invasion and the access of placenta to the maternal blood supply through suppressing effector cell immunity, inhibiting inflammation and supporting maternal vascular remodeling, thereby contributing to the maintenance of successful pregnancy (9). DCs, regulated by dramatically fluctuating maternal hormones, control the delicate balance between immune response and tolerance via intimate interactions with other immune cells (10).

Chemokines were recognized originally for their ability to dictate the migration and activation of immune cells. Many chemokines and their receptors have been identified at the MFI (11). The G-protein coupled cysteine-X-cysteine (C-X-C) chemokine receptor type 4 (CXCR4) belongs to the C-X-C family and is involved in various biological processes, including leukocyte adhesion and transendothelial migration, cellular proliferation, differentiation, apoptosis, angiogenesis, and immune regulation (12). The cognate ligand for CXCR4 is C-X-C motif chemokine ligand 12 (CXCL12) and their interaction is crucial for regulating the retention of hematopoietic stem/progenitor cells within the BM, their maintenance, and subsequent mobilization from the BM niche (13–16). CXCR4 is expressed in a variety of tissues and cell types, including neutrophils, monocytes, B cells, T cells, endothelial cells, epithelial, and stromal cells (17). Global deletion of CXCR4 or CXCL12 is embryonic lethal with severe abnormalities in BM formation, cardiovascular development, and the immune system (18, 19). Inducible ablation of CXCR4 in adult mice showed that CXCL12/CXCR4 chemokine signaling is essential for NK and DCs development and function (20, 21).

Accumulating evidence suggests that the CXCL12/CXCR4 axis plays an important role at the MFI in humans and other mammalian species (22). The CXCR4 protein is localized in stromal cells, vascular endothelial cells, and immune cells in the endometrium (23). In sheep and pigs, endometrial CXCR4 expression is upregulated during implantation and placentation (23, 24). In human endometrium, CXCR4 expression is elevated in the midsecretory phase (periimplantation) of the menstrual cycle and further increased in the decidua of early pregnancy (25). Additionally, CXCR4 expression was greater in early decidua compared with term placenta, suggesting that CXCR4 plays an important role during early placental development (26). However, the functional importance of CXCR4 at the MFI as related to implantation and pregnancy remains to be elucidated. Here, we report that global genetic ablation of CXCR4 in adult female mice, but not uterine-specific CXCR4 ablation, results in reproductive dysfunction characterized by placental developmental abnormalities, pregnancy loss, and small litter size. We show that these reproductive abnormalities are mediated via immune cell dysfunction and could be rescued by adoptive BM transplant (BMT).

## Results

### Increased resorptions and reduced litter size in CXCR4-KO mice.

**Figure 1:**
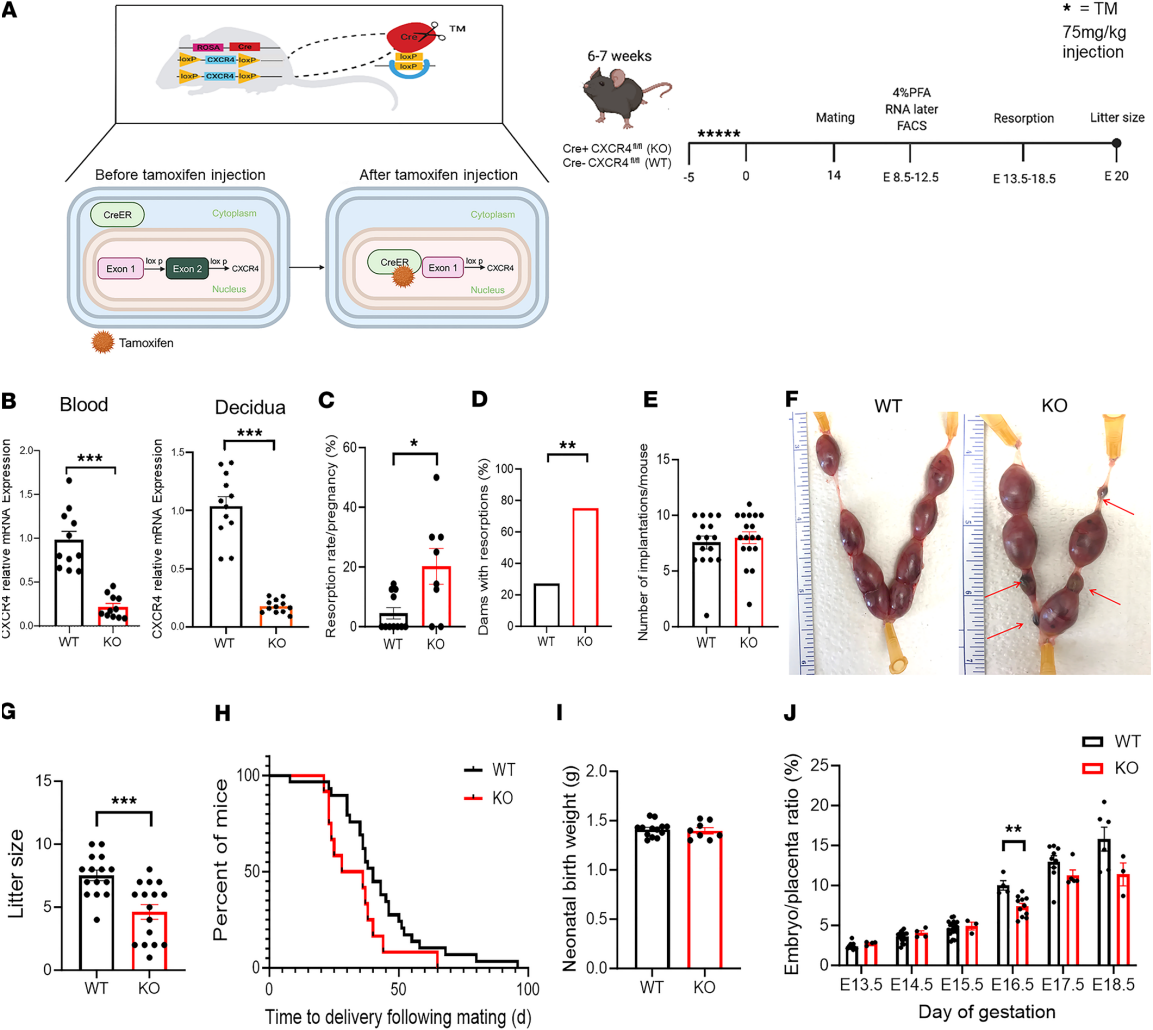
Increased resorptions and reduced litter size in CXCR4 knockout mice. (**A**) Transgenic tamoxifen-inducible Cre^+^ (CAG-Cre-ESR1) mice homozygous for the floxed CXCR4 gene were created. Upon tamoxifen administration, Cre recombinase translocates to the nucleus where it excises exon 2 of the CXCR4 gene. Cre^+^CXCR4^fl/fl^ and Cre^–^CXCR4^fl/fl^ female mice were injected with tamoxifen (75 mg/kg) over 5 days to induce Cre recombination. Following tamoxifen, Cre^+^CXCR4^fl/fl^ mice and Cre^–^CXCR4^fl/fl^ mice are designated CXCR4 KO and WT, respectively. Following a 14-day washout period, females were mated with WT males. Mice were euthanized between E9.5 and E12.5, and implantation sites were extracted for downstream analyses, or between E13.5 and E18.5 for analysis of implantation sites and resorptions. Another cohort was followed for litter size and newborn weights. (**B**) CXCR4 relative mRNA expression (RT-PCR) in peripheral blood and decidua of WT and KO mice. *n* = 10–11/ group. (**C**) Resorption rate per pregnancy (%) for WT and KO mice. *n* = 8–11/group. (**D**) Percentage of dams with resorptions in WT and KO mice. (**E**) Total number of implantation sites per pregnant mouse in WT and KO mice. *n* = 15–16/group. (**F**) Representative images of implantation sites in WT and KO mice showing resorptions (red arrows) in KO mice. (**G**) Litter size of WT and KO mice (*n* = 15–16/group). (**H**) Kaplan-Meier curve showing the time (days) from beginning of mating to delivery for WT and KO mice. (**I**) Mean neonatal birth weight/litter (grams) for WT and KO mice. *n* = 15–16 litters/group. (**J**) Ratio of embryo/placenta weight from E13.5 to E18.5 for WT and KO mice. Data shown are means of the mean embryo/placenta ratios of each pregnant dam. *n* = 15–16 mice/group. *P < 0.05; **P < 0.01; ***P < 0.001 by 2-tailed Student’s *t* test for comparisons between 2 groups. Graphical data are shown as mean ± SEM.

To study the role of maternal CXCR4 in implantation and pregnancy, we generated mice that have inducible CXCR4 gene KO using the Cre-LoxP system (Cre^+^CXCR4^fl/fl^) (Figure 1A), as we previously described (27). We used tamoxifen-inducible (TM-inducible) KO system to induce the CXCR4-KO in adulthood since embryonic CXCR4 KO results in embryonic lethality (28, 29). The CXCR4-KO mice also harbor the GFP transgene allowing us to track any transplanted donor BM cells from these mice in subsequent experiments. All experiments were initiated following a 14-day washout period from the last dose of TM administration. Successful ablation of CXCR4 gene following TM administration in Cre^+^CXCR4^fl/fl^ mice was confirmed by real-time PCR (RT-PCR), demonstrating significantly decreased CXCR4 mRNA expression in peripheral blood cells and decidua as compared with Cre^–^CXCR4^fl/fl^ littermates (*P* < 0.001; Figure 1B). Following TM administration, Cre^+^CXCR4^fl/fl^ mice and Cre^–^CXCR4^fl/fl^ mice are designated CXCR4-KO and WT, respectively. Following timed mating, CXCR4-KO mice had increased pregnancy resorptions (*P* < 0.05; Figure 1, C and F) and greater proportion of dams with resorptions (*P* < 0.01; Figure 1D), but the total number of implantation sites were not different compared with WT mice (Figure 1E). Litter size in CXCR4-KO mice was significantly smaller than WT mice; litter size was 7.5 ± 1.6 (*n* = 15) versus 4.6 ± 2.3 (*n* = 16) in WT and CXCR4-KO, respectively (*P* < 0.001; Figure 1G). The time from beginning of mating to delivery (Figure 1H) and the neonatal birth weight (Figure 1I) were not different between CXCR4-KO and WT mice. Moreover, the ratio of embryo/placenta weight in CXCR4-KO mice was smaller compared with WT mice on E16.5, but there were no significant differences at later gestational time points (Figure 1J), indicating a transient intrauterine growth restriction effect and reflecting placental dysfunction in CXCR4-KO mice. These results indicate that maternal CXCR4 deficiency led to reproductive dysfunction manifested by increased resorptions, reduction in litter size, and associated effects on placental development. To determine whether the reproductive dysfunction observed in CXCR4-KO mice was a result of CXCR4 gene ablation in maternal uterine cells or due to gene ablation in other cells, we generated conditional KO mice in which the floxed alleles of CXCR4 are coexpressed with Cre recombinase under the regulation of progesterone receptor (PGR) promoter, as we previously described (30). We confirmed that uterine CXCR4 mRNA expression is significantly decreased in PGRCre^+^/CXCR4^fl/null^ mice but not their PGRCre^–^/CXCR4^fl/wt^ littermates by RT-PCR (Supplemental Figure 1; supplemental material available online with this article; https://doi.org/10.1172/jci.insight.172216DS1). Following mating, these PGRCre^+^/CXCR4^fl/null^ mice had normal litter sizes and mean litter weights compared with control PGRCre^–^/CXCR4^fl/wt^ littermates (Supplemental Figure 1), indicating that the reproductive dysfunction observed in CXCR4-KO mice was originating from CXCR4 deficiency in exogenous extrauterine cells rather than CXCR4 deficiency in local uterine cells.

### Immune cell abnormalities in hematopoietic organs and MFI of CXCR4-KO mice.

**Figure 2:**
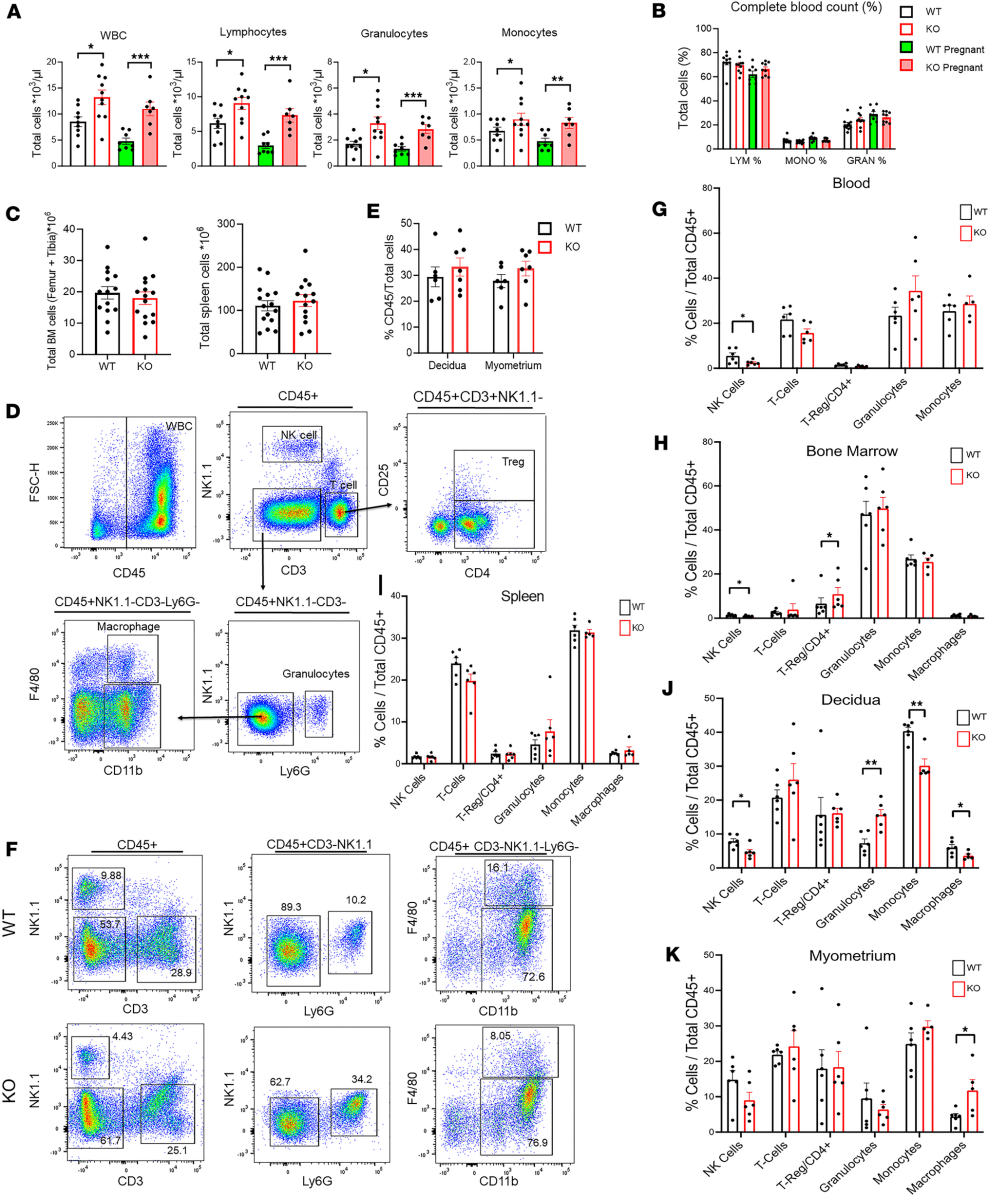
Immune cell abnormalities in hematopoietic organs and maternal-fetal interface of CXCR4-KO mice. (**A**) Total WBC, lymphocytes, granulocytes, and monocytes in peripheral blood for WT and KO pregnant (E9.5–E12.5) and nonpregnant mice. *n* = 8–10 mice/group. (**B**) Complete blood count of peripheral blood showing percentage of cell distribution for lymphocytes, monocytes, and granulocytes in WT and KO pregnant (E9.5–E12.5) and nonpregnant mice. *n* = 8–10 mice/group. (**C**) Total BM and spleen cells for WT and KO mice. *n* = 14–15 mice/group. (**D**) Gating strategy used for flow cytometry analysis of blood,a single spleen, decidua, and myometrium tissues. Representative images shown are of a spleen sample. Leukocytes were gated as CD45^+^ cells and further gated as NK1.1^+^ (NK cells) or CD3^+^ (T cells). Tregs were classified as CD3^+^CD4^+^CD25^+^ cells. Granulocytes were identified as Ly6G^+^ cells that were NK1.1^–^CD3^–^. Ly6G^–^CD3^–^NK1.1^–^ cells were gated on CD11b^+^ cells to identify CD11b^+^F4/80^+^ macrophages and CD11b^+^F4/80^–^ monocytes. (**E**) Comparison of percentage CD45^+^ cells out of total tissue cells in the decidua and myometrium of WT and KO mice. *n* = 6–7 mice/group. (**F**) Representative graphs of flow cytometry analysis of decidua from WT and KO pregnant mice (E9.5–E12.5). (**G**–**K**) Graphs summarizing the flow cytometry results showing the proportions of indicated immune subpopulations in blood (**G**), BM (**H**), spleen (**I**), decidua (**J**), and myometrium (**K**) of WT and KO pregnant mice (E9.5–E12.5). *n* = 5–6 mice/group. *P < 0.05; **P < 0.01; ***P < 0.001 by 2-tailed Student’s *t* test for comparisons between 2 groups. Graphical data are shown as mean ± SEM.

Given the well-known effects of CXCR4 KO on the hematopoietic system (20, 21) and the importance of immune cells for proper MFI development, we next wished to investigate the effects of maternal CXCR4 genetic ablation on immune cells within hematopoietic organs and the MFI. Peripheral complete blood counts (CBC) of pregnant and nonpregnant CXCR4-KO and WT mice demonstrated that the absolute numbers of total WBCs, lymphocytes, granulocytes, and monocytes were all significantly increased both in nonpregnant and pregnant CXCR4-KO mice as compared their WT mice counterparts (Figure 2A). There were no differences, however, in the relative proportions of circulating lymphocytes, granulocytes, and monocytes between the CXCR4-KO and WT groups (Figure 2B). In contrast to the gross abnormalities in absolute blood cell counts, there were no differences in absolute number of cells in the BM and spleen between the CXCR4-KO and WT groups (Figure 2C). We next analyzed immune cells in BM, spleen, and blood as well as gestational uterine tissues (decidua and myometrium) from gestation day E9.5–E12.5 by multicolor flow cytometry. The flow cytometry gating strategy is shown in Figure 2D. Among immune cell populations (CD45^+^) in peripheral blood, the proportion of NK cells was profoundly decreased in CXCR4-KO as compared with WT mice (Figure 2G). In BM, NK cells were also significantly decreased while the proportion of Tregs/CD4^+^ cells was significantly increased in CXCR4-KO mice (Figure 2H). In the spleen, there were no significant differences in immune cell proportions between CXCR4-KO and WT mice (Figure 2I). When comparing the uterine implantation site decidua and myometrium tissues between CXCR4-KO and WT mice, there were no differences in the proportions of CD45^+^ leukocytes out of total cells in either tissue (Figure 2E). In decidua, the proportions of NK cells, macrophages, and monocytes were significantly decreased, while granulocytes were significantly increased in CXCR4-KO mice (Figure 2, F and J). In myometrium, reverse trends were observed in myeloid populations with increased proportions of macrophages in CXCR4-KO mice versus WT mice (Figure 2K).

To gain further molecular insights into the immune cell aberrations that may underlie the pregnancy loss in CXCR4-KO mice, we investigated gene expression in the implantation site decidual tissue on gestational day E8.5–E12.5 by quantitative PCR (qPCR). This is a critical time of decidualization and placental formation in which NK cells are the most abundant immune cells in the decidua, playing a major role in these processes (4). The genes assayed included CXCR4/CXCL12/chemoattractants (CXCR4, CXCL12, CXCL1, CCL2, CCL5, Csf2), major inflammatory genes (*Ifng*, *Nfkb*, *Tnfa*, *Il8*, *Il10*), NK cell effector molecules (Gzmb, Gzmd, Gzme, Gzmf, Gzmg, Prf1, Klrd1, Klre1, Klrg1), and angiogenesis pathway genes (*Hif1α*, *Vegf*, *Angpt2*).

**Figure 3:**
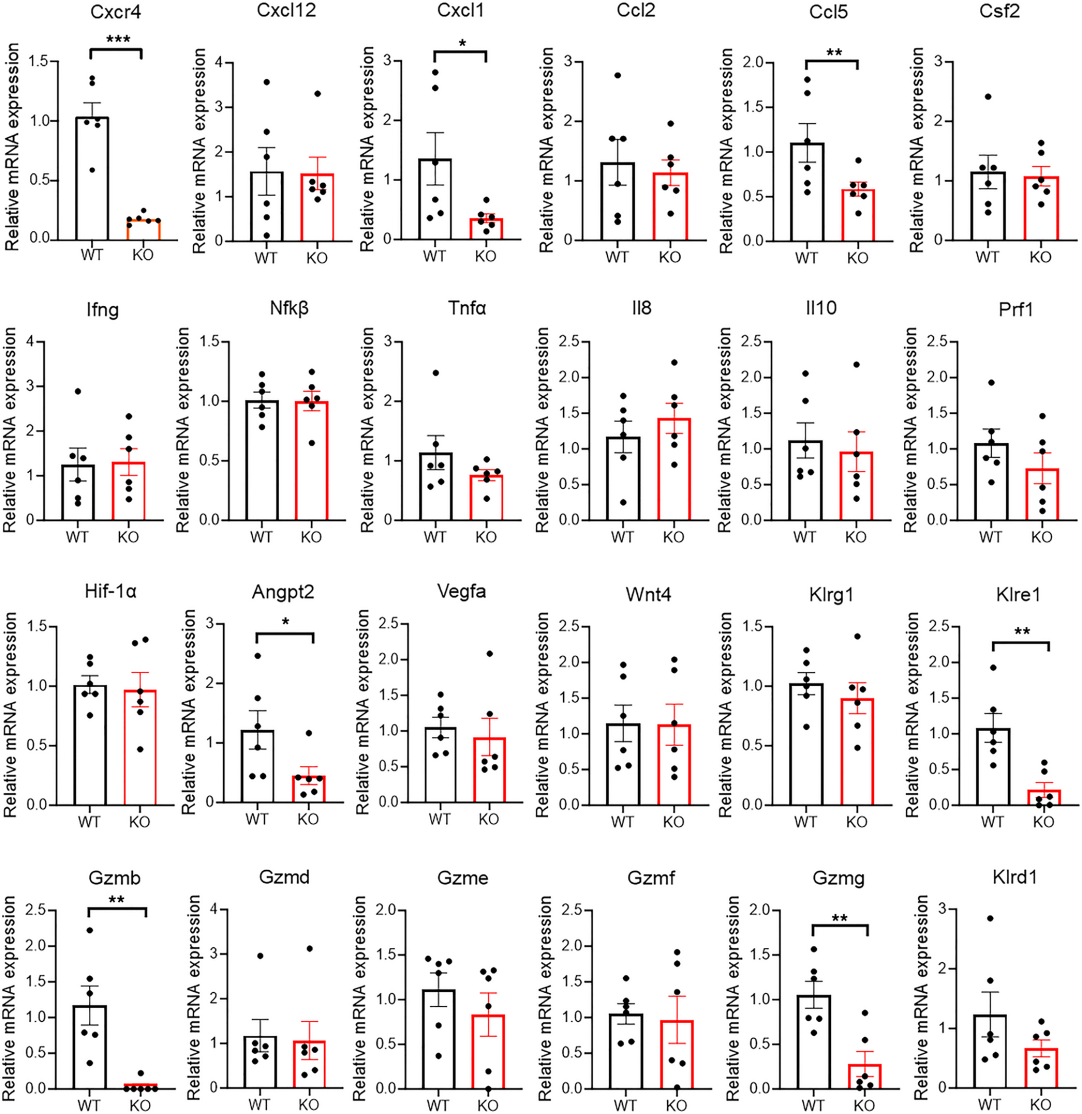
Gene expression in decidua of WT and CXCR4-KO mice. RT-PCR results showing relative mRNA expression in decidua of WT and KO mice for the indicated genes related to chemotaxis (Cxcr4, Cxcl12, Cxcl1, Ccl2, Ccl5, Csf2), inflammatory angiogenesis pathway (Hif1α, Angpt2, Vegfa), NK cell effector molecules (Klrd1, Klre1, Klrg1, Gzmb, Gzmd, Gzme, Gzmf, Gzmg, Prf1), and inflammatory pathway (*Ifng*, *Nfkb*, *Tnfa*, *Il8*, *Il10*). *n* = 5–6 mice/group. *P < 0.05, **P < 0.01, ***P < 0.001 by 2-tailed Student’s *t* test for comparisons between 2 groups. Graphical data are shown as mean ± SEM.

The decidua of CXCR4-KO mice was characterized by decreased expression of NK cytotoxic molecule Gzmb, Gzmg, and Klre1 (Figure 3), indicating dysfunction in NK cells. In addition, the neutrophil chemoattractant CXCL1 (31) and the T cell chemoattractant CCL5 (RANTES) (32) were decreased in CXCR4-KO decidua. There was no increase in acute inflammatory markers (*Ifng*, *Tnfa*, *Nfkb*). Moreover, expression of Angpt2, a central angiogenic factor that plays a key role in mouse uterine decidualization (33), was decreased in CXCR4-KO mice. To characterize gene expression profiles of immune cell populations and confirm that gene expression of specific cytotoxic molecules in the implantation sites is restricted to NK cells, we performed single-cell RNA-Seq (scRNA-Seq). Analysis on a total of 12,738 cells from E10.5 implantation sites of WT mice (*n* = 2 mice) by graph-based clustering method and guided by established markers identified all major cell clusters visualized by Uniform Manifold Approximation and Projection (UMAP) (Supplemental Figure 4, A and B). Gene expression of the cytotoxic molecules Gzmb, Gzmg, and Klre1 was demonstrated to be specific to NK cell subset (Supplemental Figure 4, C and D).

### NK cells show abnormal distribution and secretory granule dysfunction in decidua of CXCR4-KO mice.

**Figure 4:**
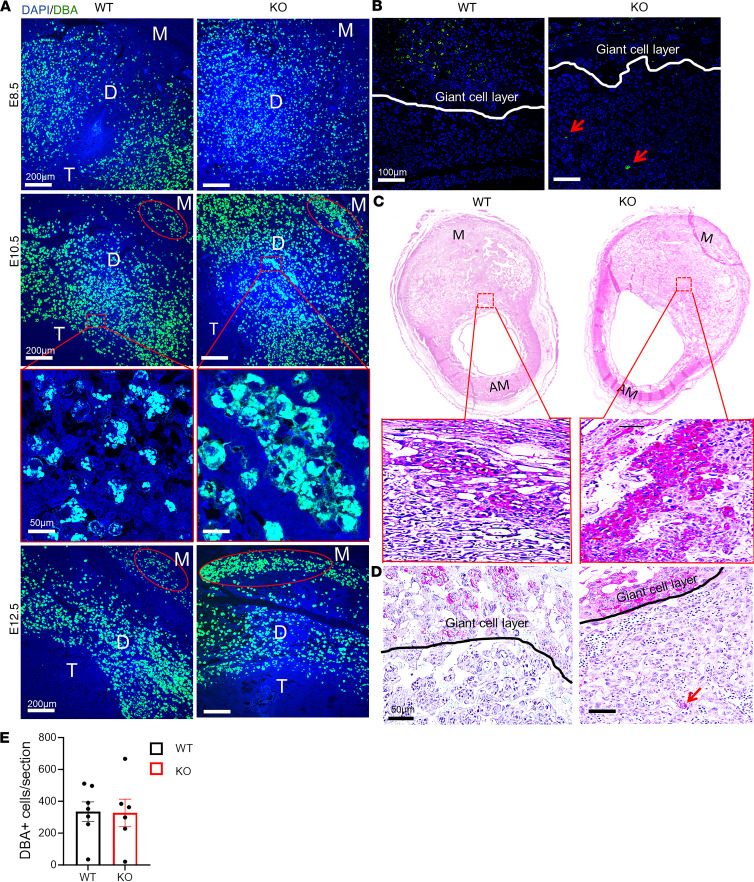
NK cells show abnormal distribution in decidua of CXCR4-KO mice. (**A**) Immunofluorescence images of decidua from WT and KO pregnant mice at E8.5, E10.5, and E12.5 showing costaining for DBA (NK cell) and DAPI (nuclei). The myometrial (M), decidua (D), and trophoblast (T) areas within the mesometrial area are shown. Note the red circle around the mesometrial lymphoid aggregate (MLAp) area demonstrating abnormal clustering of NK cells in KO decidua. Scale bars: 200 μm (low-magnification images), 50 μm (high-magnification images). (**B**) Immunofluorescence images of decidua from WT and KO pregnant mice at E10.5 coimmunostained with DBA (NK cell) and DAPI (nuclei) revealing the abnormal location of DBA^+^ NK cells beyond the giant cell layer (white line) and into the embryonic trophoblast layer in KO decidua. Scale bar: 100 μm. (**C**) Histological sections of E9.5 implantation site with PAS immunostaining showing PAS^+^ NK cells within the mesometrial area of decidua from WT and KO mice. The bottom image of each panel is a higher magnification of the dashed red rectangular area in the image above it. Scale bar: 100 μm. M, mesometrial; AM, Antimesometrial. (**D**) Higher magnification of the images in **C**. Note the presence of abnormally located PAS^+^ NK cell (red arrow) within the trophoblast (embryonic) region of the placenta past the giant cell border (black line). Scale bar: 50 μm. (**E**) Quantitation of the percentage of DBA^+^ cells per histological section in decidua of WT and KO mice. *n* = 6–7 mice/group. In **E**, there was no significant difference by 2-tailed Student’s *t* test for comparisons between 2 groups. Graphical data are shown as mean ± SEM.

**Figure 5:**
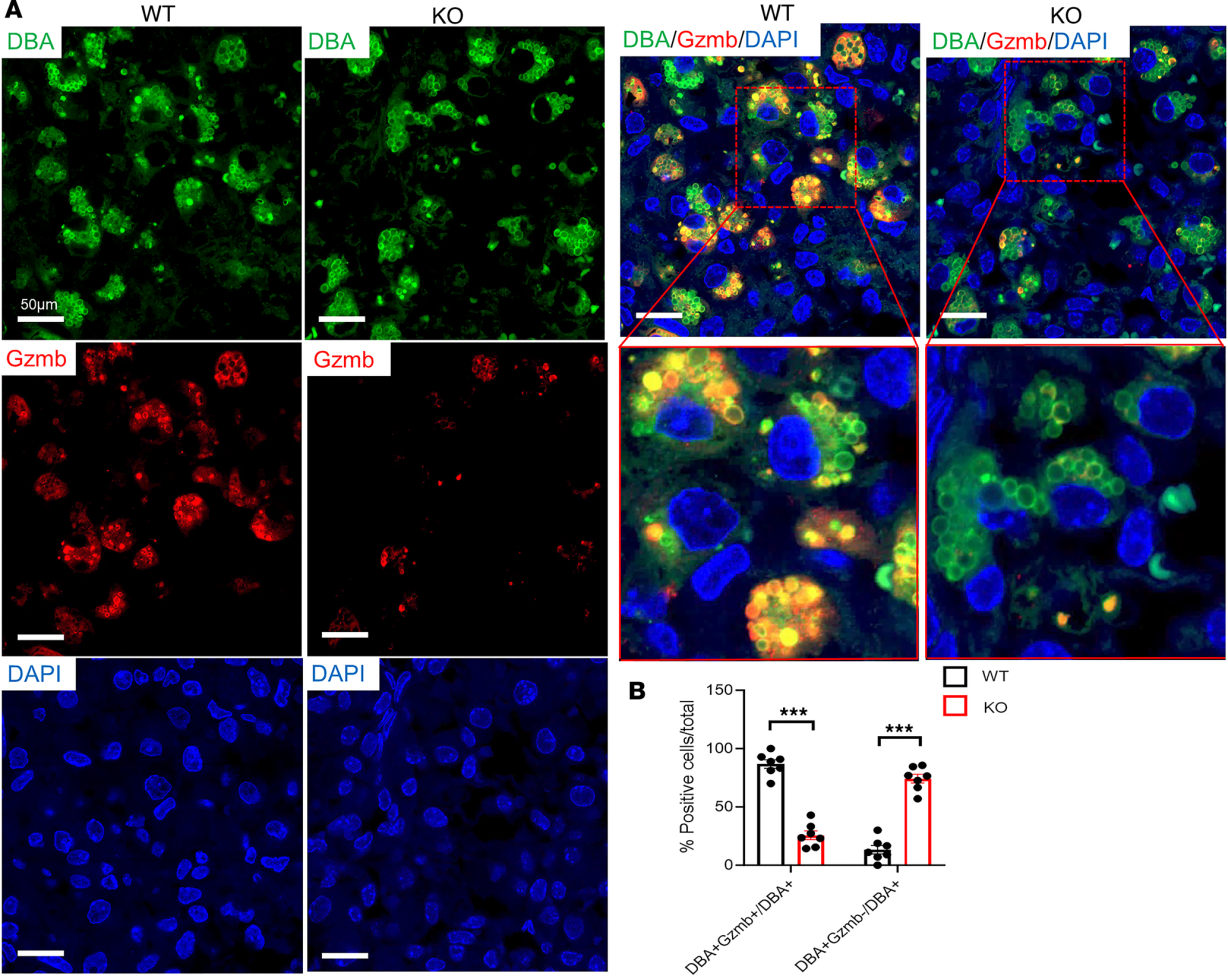
Decidual NK cells show secretory granule dysfunction in CXCR4-KO mice. (**A**) Immunofluorescence images of E10.5 decidua from WT and KO mice showing coimmunostaining for DBA (NK cell, green), Gzmb (red), and DAPI (nuclei, blue). The bottom images on the right panel are higher magnification of the dashed red areas. Note that Gzmb immunostaining normally colocalizes within secretory granules of DBA^+^ decidual NK cells, and also note the marked paucity of Gzmb immunostaining in decidual NK cells of KO mice. Scale bar: 50 μm. (**B**) Quantitation of the percentage of DBA^+^Gzmb^+^ NK cells out of total DBA^+^ NK cells and of DBA^+^Gzmb^–^ NK cells out of total DBA^+^ NK cells in decidua of WT and KO mice. *n* = 4–5 mice/group. ***P < 0.001 by 2-tailed Student’s *t* test for comparisons between 2 groups. Graphical data are shown as mean ± SEM.

Our flow cytometry results and gene expression analysis pointed to decidual NK (dNK) cell abnormalities in CXCR4-KO mice. NK cells are the most abundant hematopoietic population in the implantation site, peaking on E8.5–E10.5 and found exclusively in the mesometrial decidual area (34), where they play important roles in maternal immune tolerance, spiral artery remodeling, and placental formation (4, 35). To characterize the distribution of NK cells in the implantation site, we performed immunostaining with dolichos biflorus agglutinin (DBA) lectin, a specific marker of NK cells. DBA lectin is the commonly used marker for mouse uterine NK cells, as it stains both cytoplasmic granules as well as the cell membrane, thus identifying also immature, agranular NK cells (36). DBA staining revealed numerous DBA^+^ NK cells infiltrating the mesometrial area of the decidua from E8.5 to E12.5 in both CXCR4-KO and WT mice (Figure 4A), and the overall number of DBA^+^ NK cells per section was no different between the 2 groups (Figure 4E). However, the mesometrial lymphoid aggregate (MLAp) region showed abnormally high concentrations of NK cells on E10.5 and E12.5 in the CXCR4-KO compared with WT group (Figure 4A). In addition, abnormal NK cell aggregates were found in other decidual areas on E10.5 and E12.5 in CXCR4-KO mice (Figure 4A). Periodic acid–Schiff (PAS) reagent is another NK-specific marker staining cytoplasmic granules of uterine NK cells. Since some populations of NK cells are DBA^–^ but PAS^+^, PAS has been shown to be more inclusive of all dNK cell populations (36). PAS staining corroborated our findings showing abnormal aggregation of PAS^+^ NK cell clusters within the decidua in CXCR4-KO mice (Figure 4C). Both DBA and PAS staining also revealed several NK cells that infiltrated past the giant cell layer (trophoblast) and invaded into the trophoblast (embryonic) area in CXCR4-KO decidua (Figure 4, B–D). This was not seen in WT mice (Figure 4, C and D). Granzyme B is a protease found in cytolytic granules of NK cells and is best known for its capacity to kill target cells (37). However, more recent reports indicate that granzyme B may also have important noncytolytic immunosuppressive functions (38). We wished to confirm our finding that Gzmb mRNA expression was greatly decreased in CXCR4-KO decidua at the protein level. Coimmunostaining of GZMB protein with DBA lectin (Figure 5A) showed a striking reduction in GZMB granule production by NK cells, with 25.6% ± 2.9% DBA^+^GZMB^+^ cells out of total DBA^+^NK cells in CXCR4-KO versus 86.7% ± 3.5% DBA^+^GZMB^+^ out of total DBA^+^NK cells in WT mice decidua (*P* < 0.01; Figure 5, A and B). The paucity of GZMB expression in dNK cells indicates their dysfunctional state, which may contribute to altered inflammatory milieu at the MFI. In addition to the NK cell population, immunostaining for the granulocyte marker Ly6G in decidua showed that neutrophils were more numerous in the CXCR4-KO decidua as compared with WT mice (Supplemental Figure 2), consistent with our flow cytometry data.

### BMT from WT donors rescues pregnancy resorption in CXCR4-KO mice.

**Figure 6:**
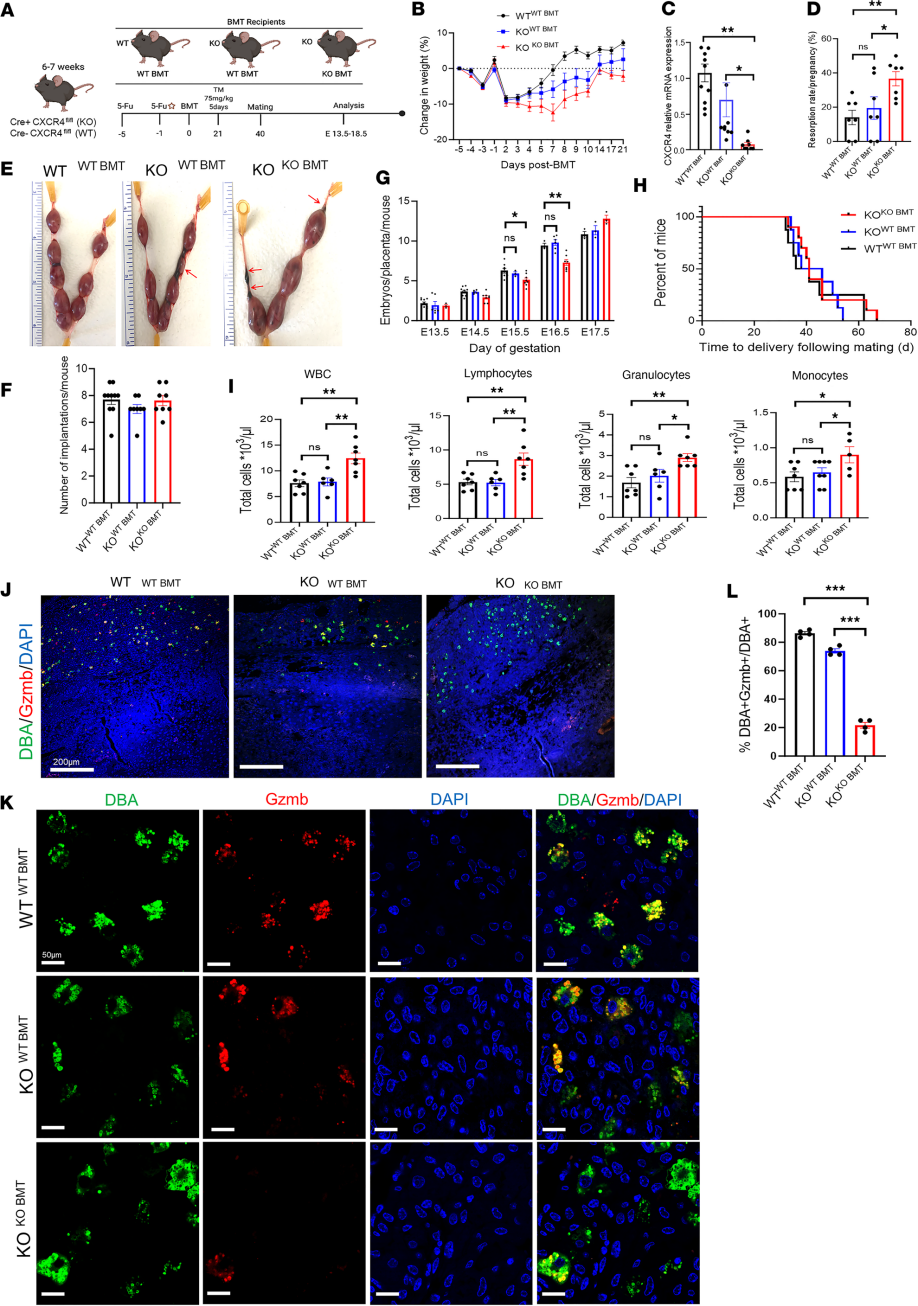
BM transplantation from WT donors rescues pregnancy loss in CXCR4-KO mice. (**A**) Six- to 7-week-old Cre^+^CXCR4^fl/f^ female (KO) mice underwent BM transplant (BMT) from either Cre^–^CXCR4^fl/fl^ (WT) or KO donor mice following our established 5-fluorouracil (5FU) protocol. Control WT female mice underwent BMT from WT donors. All 3 mice groups received submyeloablation with 5FU doses on days –5 and –1 before their BMT. Twenty-one days after their BMT, the mice received injections of tamoxifen (TM) (75 mg/kg). Timed pregnancies were established, with WT male mice starting 40 days after their BMT, and mice were euthanized for tissue analysis between E13.5–E18.5. (**B**) Percent weight change in WT^WT–BMT^ mice, KO^WT–BMT^ mice, and KO^KO–BMT^ mice in the period preceding and following the BMT and prior to the TM injections. *n* = 9–12/group. (**C**) RT-PCR showing CXCR4 relative mRNA expression in peripheral blood. *n* = 8–11/group. (**D**) Resorption rate per pregnancy percentage. *n* = 7/group. (**E**) Images of E13.5–E14.5 uteri. Note red arrows pointing to resorptions in KO^WT–BMT^ mice and KO^KO–BMT^ mice. (**F**) Total number of implantation sites per pregnant mouse. *n* = 8–10/group. (**G**) Ratio of embryo/placenta weight per mouse on E13.5, E14.5, E15.5, E16.5, and E17.5. *n* = 3–8/time point. (**H**) Kaplan-Meier curve showing time to delivery from the start of mating. *n* = 8–10/group. (**I**) Complete blood count (CBC) of peripheral blood revealing levels of WBC, lymphocytes, granulocytes, and monocytes. *n* = 6–7/group. (**J** and **K**) Immunofluorescence sections of decidua from WT^WT–BMT^, KO^WT–BMT^, and KO^KO–BMT^ mice. Coimmunostaining with DBA (green), Gzmb (red), and DAPI (blue) is shown. Scale bars: 200 μm (low-magnification images; **J**) and 50 μm (high-magnification images; **K**). Note abundant colocalization of DBA^+^ NK cells (green) with GZMB (red) in decidua of WT^WT–BMT^ and KO^WT–BMT^ mice, but not in KO^KO–BMT^ mice. (**L**) Quantitation of percentage of DBA^+^Gzmb^+^ NK cells out of total DBA^+^ NK cells in WT^WT–BMT^, KO^WT–BMT^, and KO^KO–BMT^ mice. *n* = 4/group. *P < 0.05, **P < 0.01, ***P < 0.001 by 1-way ANOVA with Sidak’s post hoc correction for multiple comparisons. Graphical data are shown as mean ± SEM.

To determine whether the reproductive dysfunction manifested by pregnancy loss in CXCR4-KO mice is mediated via immune cell dysfunction, we performed adoptive BMT of CXCR4-expressing WT donor cells or CXCR4-KO donor cells into CXCR4-KO recipient mice. For this end, we utilized our previously described nongonadotoxic 5-fluorouracil–based (5FU–based) submyeloablation protocol that preserves fertility in BM recipient mice (39, 40). BMT was performed from WT donors into KO mice (KO^WT-BMT^), and KO donors into KO (KO^KO-BMT^). WT mice receiving BMT from WT donors (WT^WT-BMT^) served as controls (Figure 6A). Following 5FU-based BMT, all 3 mice groups exhibited weight decrease followed by rapid recovery (Figure 6B) as previously described with this protocol (39, 40). Successful BM engraftment was confirmed by RT-PCR analysis of peripheral blood on day 21 after BMT, demonstrating that peripheral blood cells of CXCR4-KO mice with BMT from WT donors had increased CXCR4 mRNA expression (65.1% expression of WT^WT-BMT^) as compared with KO^KO-BMT^ (7.1% expression of WT^WT-BMT^, *P* < 0.01; Figure 6C). We found that pregnancy loss in CXCR4-KO mice was partially rescued by BMT from WT donors. The resorption rate was comparable between KO^WT-BMT^ mice and WT^WT-BMT^ control mice but was significantly increased in KO^KO-BMT^ compared with WT^WT-BMT^ (*P* < 0.05; Figure 6, D and E). The total number of implantation sites, however, was no different between the groups (Figure 6F). The transient abnormally decreased ratio of embryo/placenta on E15.5 and E16.5 was also normalized in CXCR4-KO mice receiving WT-BMT (Figure 6G). There was no difference in the time from mating to delivery between the 3 groups (Figure 6H). Moreover, complete peripheral blood counts showed that the absolute numbers of total WBCs, lymphocytes, granulocytes, and monocytes were decreased in both KO^WT-BMT^ and WT^WT-BMT^ mice as compared with KO^KO-BMT^ (Figure 6I), indicating normalization of peripheral blood cell parameters in CXCR4-KO mice following WT-BMT.

To determine whether the abnormalities in NK cell granules in CXCR4-KO mice were mitigated by the BMT from WT donors, we analyzed the decidua for colocalization of GZMB and DBA, an NK-specific marker. We found greater abundance of DBA^+^Gzmb^+^ NK cells out of total DBA^+^ NK cells in decidua of KO^WT-BMT^ mice (73.9%) than in KO^KO-BMT^ mice (21.6%) (Figure 6, J–L). Colocalization of DBA and GZMB with GFP, which labels donor BM–derived cells, showed that the GFP^+^ BM–derived NK cells were the main source of GZMB protein in the decidua of KO^WT-BMT^ mice (Supplemental Figure 3), indicating that the increase in GZMB expression in the rescued CXCR4-KO^WT-BMT^ mice originated from the transplanted hematopoietic cells.

### Placental abnormalities in CXCR4-KO mice are rescued by BMT from WT donors.

**Figure 7:**
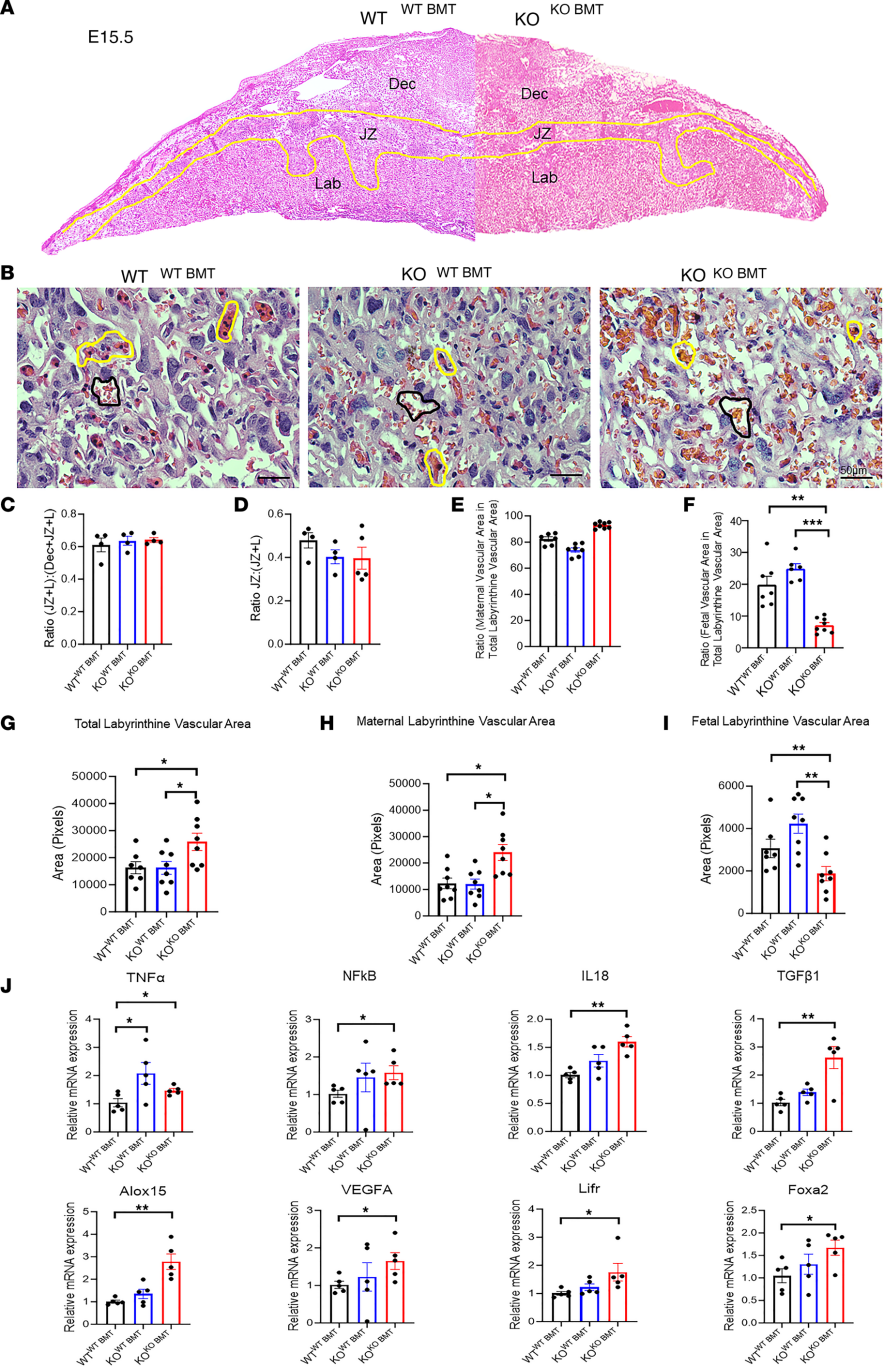
Placental abnormalities in CXCR4-KO mice are rescued by BM transplantation from WT donors. (**A**) Histological sections of E15.5 placenta in WT^WT–BMT^ mice on the left, and KO^KO–BMT^ on the right. Areas of decidua (Dec), junctional zone (JZ), and labyrinth (Lab) are demarcated by the yellow lines. (**B**) Microscopic images of placental labyrinthine zone in WT^WT–BMT^, KO^WT–BMT^, and KO^KO–BMT^ mice. Note maternal vascular space demarcated by black line and the fetal vascular space, containing nucleated RBCs, demarcated by yellow lines. Scale bar: 50 μm. (**C**–**F**) Ratio of placental areas of JZ + L/Dec + JZ + L (**C**), JZ/JZ + L (**D**), maternal vascular area/total labyrinthine vascular (**E**), and fetal vascular area/total labyrinthine vascular area (**F**). *n* = 4–8 mice/group. (**G**–**I**) Placental labyrinthine areas (pixels) of WT^WT–BMT^, KO^WT–BMT^, and KO^KO–BMT^ mice showing total labyrinthine vascular (**G**), maternal labyrinthine vascular area (**H**), and fetal labyrinthine vascular area (**I**). *n* = 7–8/group. (**J**) RT-PCR showing relative mRNA expression of specified genes in placental tissues. *n* = 4–5/group. *P < 0.05, **P < 0.01, ***P < 0.001 by 1-way ANOVA with Sidak’s post hoc correction for multiple comparisons. Graphical data are shown as mean ± SEM.

Since dNK cells have been associated with modulation of placental development and vasculature during early pregnancy and midpregnancy (4, 41), our next aim was to characterize the structural changes in placental development. Histopathological analysis of placental areas on E15.5 showed no gross morphological differences between WT^WT-BMT^, KO^WT-BMT^, and KO^KO-BMT^ mice groups (Figure 7A). Placental morphometric analysis demonstrated that the proportional depth of the placental disc including the labyrinth (L) and junctional zone (JZ) relative to the decidua (Dec), expressed as (JZ + L)/(Dec + JZ + L), and the proportion of the junctional zone relative to the labyrinth, expressed as JZ/(JZ + L), did not differ among the 3 groups (Figure 7, B–D). To investigate differences in placental vascularity, we focused on the labyrinth zone, which consists of cells of trophectodermal and mesodermal origin that together undergo branching morphogenesis, providing a large surface area for gas and nutrient exchange between the mother and fetus. Interestingly, there were significant placental vascular abnormalities in the CXCR4-KO^KO-BMT^ as compared with CXCR4-KO^WT-BMT^ and WT^WT-BMT^ control mice. The CXCR4-KO^KO-BMT^ animals had greater overall total labyrinthine vascular area, greater maternal vascular spaces, and less embryonic vascular spaces in the labyrinthine zone as compared with both CXCR4-KO^WT-BMT^ and control mice (Figure 7, G–I). Moreover, the ratio of fetal vascular area to total labyrinthine vascular area was decreased in CXCR4-KO^KO-BMT^ as compared with CXCR4-KO^WT-BMT^ and control mice (Figure 7F). There was no difference in the ratio of maternal vascular area to total labyrinthine vascular area between the groups (Figure 7E). These data indicate that maternal CXCR4 expression plays a role in normal placental vascular development and that BMT of CXCR4-expressing hematopoietic cells can reverse placental vascular abnormalities in CXCR4-KO mice.

To expand on these findings, we investigated gene expression alterations in placental tissues from the same 3 experimental groups. We found that the expression of central inflammatory genes including *Tnfa*, *NfκB*, and *Il18* were significantly increased in CXCR4-KO^KO-BMT^ as compared with control mice. Moreover, the expression of key genes in placental development (Foxa2, Lifr) (42, 43), placental vascular development (Vegf, Alox15) (44, 45), and trophoblast invasion (TGFβ1) (46) were also significantly increased in KO^KO^ ^BMT^ as compared with control mice (Figure 7J).

## Discussion

In this work, we report that adult-induced maternal global genetic CXCR4 deletion, but not uterine-specific CXCR4 deletion, leads to pregnancy loss associated with dNK cell dysfunction and placental vascular abnormalities. Moreover, we show that adoptive BM transfer of WT CXCR4^+^ BM cells into CXCR4-deficient mice was able to rescue the reproductive deficits along with normalizing dNK cell function and placental vascular development. Taken together, these data indicate that pregnancy loss and placental abnormalities in our model were likely related to immune cell dysfunction mediated by CXCR4 ablation in hematopoietic cells.

Maternal adaptations to pregnancy are critical during mammalian development. It is well established that immune-competent cells in the decidua are important for placental development and embryo survival. In this study, genetic ablation of CXCR4 resulted in increased absolute leukocyte numbers in peripheral blood, consistent with the known role of the CXCR4/CXCL12 axis in retention of hematopoietic cells within the BM (47–49). In our study, we observed alterations in proportions of multiple immune cell populations in hematopoietic organs and decidua of CXCR4-KO mice. Specifically, the proportions of NK cells, macrophages, and monocytes were significantly decreased, while granulocytes were significantly increased in decidua of CXCR4-KO mice. In agreement, we have previously shown that genetic ablation of CXCR4 in adult BM cells — including NK cells, monocytes, macrophages and granulocytes — affects their recruitment to the mouse implantation site (27). Moreover, the CXCL12/CXCR4 axis has been reported to be important for recruitment of NK cells (50) and DCs (51) to decidua. In contrast to myeloid and NK cell subpopulations that were altered in hematopoietic organs and decidua in our study, T cell numbers were unaffected. This is also consistent with prior studies showing that CXCR4 deletion does not impair T cell precursors and does not influence localization of T lymphocytes in secondary lymphoid organs (21, 49).

dNK cells become abundant in mice during decidualization as they mediate important functions at the MFI; they produce cytokines and support trophoblast invasion and spiral artery remodeling, which are crucial for successful placentation and pregnancy (4, 5, 7). Prior studies have demonstrated that the CXCR4/CXCL12 axis is essential for NK cell development and maturation in Mx1-Cre–generated CXCR4-deficient mice (21). Mice with inducible deletion of CXCR4 in adulthood have reduced numbers of mature NK cells in their BM and spleen. Moreover, mature NK cells from mice with this CXCR4 deletion have reduced expression of perforin, a key effector molecule of cell-mediated cytotoxicity (21). Consistent with these prior findings, in our study, NK cell numbers were also decreased in CXCR4-KO mice as observed in peripheral blood, BM, and decidua. Moreover, we found that NK cells in decidua of CXCR4-KO mice showed abnormal clustering and distribution and profoundly decreased expression of granzyme B in their secretory granules, indicating NK cell–maturation defects.

Mature NK cells classically interact with target cells to form immune synapses and release perforin and granzymes through exocytosis, killing target cells directly (52). The protease granzyme B is a mediator of NK-related cytotoxicity and is best known for its capacity to kill target cells. This cytotoxicity occurs after its intracellular delivery via cleavage and activation of proapoptotic caspases (37). Interestingly, more recent reports provide evidence that granzyme B may also have noncytotoxic functions. Granzyme B cleaves extracellular matrix components and enables chemokine-driven movement of lymphocytes through basement membranes (53). Moreover, GZMB-null NK cells have defective cell trafficking and an inability to efficiently cross blood vessel walls (53). Thus, the dramatic decrease of granzyme B expression observed in dNK cells in our study may explain our findings of decreased numbers and abnormal clustering of NK cells in decidua of CXCR4-KO mice. NK cell ablation in mice has been shown to result in pregnancy loss associated with increased expression of genes related to inflammation and immune activation, suggesting that NK cells prevent the development of alloimmunity during normal pregnancy by modulating the immune-regulatory functions of DCs during early gestation (54). Moreover, secretion of IL-10 is likely an important signal promoting this NK-induced immune tolerance as studies in IL10^–/–^ mice have shown an increased susceptibility to inflammatory insults leading to pregnancy loss due to excessive infiltration and cytotoxic activation of uterine NK cells (55, 56). In addition, it was recently shown that NK cell–mediated granzyme B is required for activation of type 2 immune response in a mouse model of asthma (38). While such a role for granzyme B at the MFI has not been previously described, it is interesting to speculate that NK cell–mediated granzyme B has a similar immunomodulatory role in pregnancy, which is characterized by a type 2 immune response. Indeed, the placentas of CXCR4-KO mice in our model had increased gene expression of several type 1 immune proinflammatory mediators including *Tnfa*, *Nfkb*, and *Il18*, consistent with an abnormally increased type 1 immune response, which may be linked to the granzyme B deficiency of dNK cells.

CXCR4 deficiency has been previously linked to increased type 1 and innate immune response (57–59). McIntosh et al. have shown that intrauterine administration of the CXCR4 antagonist AMD3100 in pregnant sheep results in decreased endometrial expression of IL-10 and Akt and increased expression of IL-12, indicating a shift to a proinflammatory type 1 immune phenotype (57). The CXCR4 antagonist AMD3100 has also been shown to lead to a similar proinflammatory response in other tissues, including lung and kidney (58, 59). In an in vitro human study, pretreatment of cocultures of decidual stromal cells and trophoblast cells with a neutralizing anti-CXCR4 antibody resulted in overexpression Th1-type cytokines IFN-γ and TNF-α in trophoblasts (60), consistent with a shift toward a type 1 immune response. Our findings are consistent with these studies, showing that CXCR4 ablation results in increased placental gene expression of proinflammatory genes (*Tnfa*, *Nfkb*, *Il18*). Taken together, these data indicate that the CXCL12/CXCR4 axis plays an important role in the shift from type 1 to type 2 immune response during pregnancy.

IL-18, a mediator of type 1 immune response, is a major neutrophil product and activator of NK cells, directly inducing NK cells to produce IFN-γ, which is a crucial effector cytokine that controls NK cell activation (61, 62). It has been shown that, in mice with specific CXCR4 deletion in myeloid cells, neutrophils are increased and display increased IL-18 expression that promotes NK cell activation (63). Our data showing increased numbers of neutrophil cells (Ly6G^+^) in decidua of CXCR4-KO mice is consistent with these results, and it is likely that they are the cell type responsible for the increased expression of IL-18 observed in CXCR4-KO placentas.

The placenta is a key organ that constitutes an interface between the maternal and fetal circulations, facilitating nutrient uptake, metabolic/gas exchange, and waste elimination. The mouse placenta consists of 3 layers: the labyrinth, comprising a highly branched fetal vascular network in close apposition to trophoblast-lined maternal sinusoids, maximizing surface area for efficient fetal-maternal exchange; the junctional zone, comprising trophoblast giant cells and spongiotrophoblast cells that invade the decidua and maternal blood vessels; and the maternal decidua, the specialized endometrium that includes decidual and immune cells and maternal blood vessels. Inadequate differentiation and/or development of these layers results in placental structural changes that can affect the supply of oxygen and nutrients to the fetus. Dysregulation of placental angiogenesis has emerged as one of the main pathophysiological features in the development of placental insufficiency and related clinical conditions, including fetal growth restriction and preeclampsia (64). In this study, the placenta of mice with maternal CXCR4 deletion showed vascular abnormalities that included greater maternal vascular spaces and decreased fetal vascular spaces in the labyrinthine zone as compared with control mice. Notably, CXCR4-KO mice in our study displayed a transient fetal growth restriction effect at late gestational age (E15.5–E16.5), but fetal weights subsequently normalized by the time birth and pup weights were normal. While these growth effects may be related to the vascular abnormalities observed, it is plausible that the growth effect may be only transient due to compensatory CXCR4 expression by fetal trophoblast tissue, given that the Cre-mediated CXCR4 ablation is restricted to maternal tissues. Moreover, the observed vascular abnormalities were associated with increased placental gene expression of VEGF. Decidual mRNA levels of VEGF were unchanged, but levels of angiopoietin-2 were downregulated in CXCR4-KO animals as compared with controls. Placental vascular abnormalities in our study could be related to the NK cell dysfunction observed. Indeed, dNK cells play a role in regulating the optimal timing and progression of decidual angiogenesis (7), and unbalanced relative NK cell abundance caused by in vivo NK cell depletion leads to placental vascular alterations with similarly decreased labyrinthine embryonic vascular spaces associated with upregulation of antiangiogenic growth factors (54, 65); this suggests that NK cells are required for early vascular responses associated with pregnancy.

VEGF and its receptors are the main drivers of placental angiogenesis, vascular growth, and maturation (66, 67). Moreover, CXCL12/CXCR4 signaling stimulates VEGF production and secretion, and in turn, VEGF upregulates CXCL12 and CXCR4 synthesis, thereby creating a powerful feed-forward loop (68–70). A prior report by Runyan et al. demonstrated that CXCR4 antagonism using intrauterine administration of AMD3100 in early pregnancy in sheep results in placental vascular abnormalities and decreased endometrial expression of VEGF (71). In their study, however, pregnancy success was similar between control and AMD3100-treated ewes. In contrast, we observed pregnancy losses in CXCR4-KO animals in our study. This discrepancy may be related to the limited duration of CXCR4 antagonism (from day 12 to 20 after breeding) in the study by Runyan et al. (71) as compared with the chronic ablation of CXCR4 throughout pregnancy in our study. A possible explanation for the increase in VEGF levels observed in our study, in contrast to findings by in ref. 71, may be related to a compensatory angiogenic response by trophoblast (fetal derived) given that Cre-mediated genetic CXCR4 ablation in our study was limited to maternal cells. In contrast, CXCR4 antagonism by intrauterine AMD3100 infusion would affect CXCR4 at the MFI in both maternal and fetal tissues. Interestingly, Runyan et al. (71) described a compensatory increase in CD34^+^ hematopoietic progenitor cells in the uterus of pregnant sheep in response to AMD3100, suggesting that this was a vascular rescue mechanism (72).

Findings in our model implicating the CXCL12/CXCR4 axis in pregnancy loss and placental developmental and vascular abnormalities may also provide insights into related pregnancy pathologies in humans. Indeed, accumulating evidence in humans suggests that dysfunction of the CXCL12/CXCR4 axis may be related to pregnancy complications, including miscarriage and preeclampsia. Low expression levels of CXCL12 and CXCR4 in peripheral blood and decidual/placental tissues are associated with miscarriage and preeclampsia in women (73–75). Moreover, polymorphisms in CXCR4 and CXCL12 have been associated with preeclampsia in humans (76).

In conclusion, our study presents compelling evidence that CXCL12/CXCR4 signaling in hematopoietic cells is central to immune cell development and function at the MFI, initiating downstream events that regulate type 2 immune response locally and helping to maintain proper placental and pregnancy development. Further studies will be needed to explore the therapeutic potential of interventions aimed at the CXCL12/CXCR4 axis in pregnancy disorders related to immunological and angiogenic imbalances during early gestation, such as recurrent pregnancy loss and preeclampsia.

## Methods

### Mouse experimental studies.

Mice were maintained in a controlled environment at the Animal Facility of Yale School of Medicine with a 12-hour light, 12-hour dark cycle with ad libitum access to food and water. To generate TM-inducible CXCR4-KO mice, we used the Cre/LoxP TM-inducible system, as we described before (27). All mice were purchased from The Jackson Laboratory. All animals had C57BL/6J background. Mice expressing the Cre recombinase gene under the control of the TM-inducible promoter (B6.Cg-Tg[CAG-Cre/Esr1*]5Amc/J, stock no. 004682) were bred with mice in which the critical exon 2 of the CXCR4 gene is flanked by 2 loxP sequences (B6.129P2-Cxcr4tm2Yzo/J, stock no. 008767) (20). The Cre^+^/CXCR4^fl/fl^ and Cre^–^/CXCR4^fl/fl^ mice were injected with 75 mg/kg TM (i.p.; Sigma-Aldrich) for 5 consecutive days. Upon TM administration, TM binds to and allows translocation of the Cre-recombinase to the nucleus, where it causes site-specific recombination of the LoxP sites resulting in KO of the CXCR4 gene (Figure 1A). For animal experiments, Cre^+^/CXCR4^fl/fl^ mice and their Cre^–^/CXCR4^fl/fl^ littermates were used. After TM treatment, we refer to Cre^–^/CXCR4^fl/fl^ mice as WT and Cre^+^/CXCR4^fl/fl^ mice as CXCR4-KO. The Cre/Lox system was also used to generate the conditional uterine-specific CXCR4-KO mice. Mice with the Cre recombinase gene under control of the PGR promoter (PgrCre^+^) were crossed to mice with floxed CXCR4 gene (CXCR4^fl/fl^) (stock nos. 017915 and 008767, respectively; The Jackson Laboratory) (30). Mice were genotyped to confirm Cxcr4^fl/fl^ homozygosity together with PgrCre^+^ transgene as described previously (30). These female PgrCre^+^/CXCR4^fl/fl^ mice were then bred with WT male mice, and litter sizes were compared. CXCR4^fl/fl^ female mice were bred with WT male mice 2 weeks after the final TM treatment and checked for vaginal plugs daily. The day of vaginal plug detection was considered E0.5. Following delivery, the litter size and pup weights were recorded, and time to delivery from initiation of mating was calculated. For the implantation experiment, CXCR4 WT and CXCR4-KO mice were bred and checked for vaginal plugs daily as described above. Mice were euthanized between E8.5 and E12.5, and implantation sites, blood, BM, and spleen tissues were extracted for flow cytometry analysis or were stored in either RNALater or 4% paraformaldehyde for subsequent analyses. For the resorption experiment, plug-positive female mice were euthanized at various gestational time points, including E13.5, E14.5, E15.5, E16.5, E17.5, and E18.5, and the ratio of embryo/placenta weight was calculated. Before euthanasia, peripheral blood was collected by retro-orbital venipuncture into EDTA-coated tubes for hematological assessment and immediately analyzed using a HemaTrue hematology analyzer (Drew Scientific) according to the manufacturer’s protocol. The uterus was inspected for presence of implantations and resorptions, and the mean resorption rate per pregnancy, the number of implantation sites per mouse, and percentage of dams with resorptions were calculated. The implantation sites were extracted for histology, IHC, immunofluorescence, and gene expression level analysis.

### TM injection.

TM (Sigma-Aldrich) was dissolved in corn oil at a concentration of 20 mg/mL. TM was administered to mice by i.p. injection for 5 consecutive days (75 mg/kg). Retro-orbital blood was obtained by venipuncture 2 weeks after the last dose of TM to assay for Cxcr4 mRNA for determination of Cxcr4 downregulation in peripheral blood cells.

### Processing of BM, spleen, blood, decidua, and myometrium by flow cytometry.

Blood, spleen, BM, decidua, and myometrium were extracted for flow cytometry following the protocol described before (40). Successfully bred female mice were killed at gestational age E8.5–E12.5 for flow cytometry to characterize immune cells. Uterine implantation sites were dissected to separate decidua and myometrium using a dissecting microscope (77). Briefly, an individual implantation site was isolated by making 2 vertical cuts along the short axis of the uterine horn and opened at its antimesometrial edge, retracting its wall mesometrially and exposing the decidual capsule. Outer uterine (myometrial) tissue was then separated from the underlying decidua and MLAp. Following separation of the myometrium, the underlying decidual capsule was opened, facilitating removal of the placenta and embryonic tissues. Dissected myometrial and decidual tissues were then minced and subsequently digested with a solution of HBSS (Invitrogen) containing collagenase B (1 mg/mL; Roche Diagnostics), deoxyribonuclease I (0.1 mg/mL; Sigma-Aldrich), and 1% penicillin/streptomycin (Thermo Fisher Scientific) antibiotics for 30 minutes at 37°C. Only implantation sites without evidence of resorption were chosen for analysis, resorptions were excluded, and 1 implantation site per mouse was analyzed separately. Cell suspension was filtered through 70 μm filter and centrifuged at 805*g* at 4°C for 8 minutes. The cell pellet was then washed with PBS, centrifuged at 805*g* for 5 minutes, and then resuspended in FACS buffer (2% FBS in PBS). BM was flushed from tibias and femur bones with DMEM/F12 medium. The whole spleen was removed and crushed using mortar and pestle to extract splenic cells. Peripheral blood was obtained by retro-orbital venipuncture and collected into EDTA-coated tubes on ice. All samples were filtered using sterile 70 μm mesh and centrifuged at 805*g* at 4°C for 5 minutes followed by RBC lysis for 10 minutes at room temperature in RBC lysis buffer as per the manufacturer’s protocol (Mltenyi Biotec). FACS buffer was added to neutralize the RBC lysis, and cell suspension was centrifuged again (805*g* at 4°C for 5 minutes). Cell suspensions were incubated with mouse Trustain FcX PLUS anti CD16/32 (BioLegend) blocking for 10 minutes, followed by incubation with the appropriate antibodies for 30 minutes on ice. The cells were then washed 3 times with FACS buffer at 453*g* at 4°C for 5 minutes, and the pellet was resuspended in 500 μL ice-cold PBS for flow cytometry analysis. Flow cytometry was performed on LSRII Green (BD Biosciences). At least 10,000 cells per sample were analyzed for myometrial tissue, and at least 100,000 cells per sample were analyzed for each of the other tissues. Gates were applied to forward-scatter/side-scatter dot plots to exclude nonviable cells and cell debris. Appropriate unstained and antibody IgG isotype controls were used for setting compensation and determining gates. Data were analyzed using the software FlowJo V10 (FlowJo). Antibodies used in flow cytometry analysis are listed in Supplemental Table 1.

### 5FU-based submyeloablation and BMT.

Submyeloablation was performed using the 5FU-based protocol in combination with stem cell factor as previously described (39). General toxicity of the treatment regimen was monitored by measuring weights of mice and checking mouse well-being daily. For the CXCR4 adoptive BMT experiments, BMT was performed from either Cre^–^CXCR4^fl/fl^ (WT) GFP transgenic male donor mice into Cre^+^CXCR4^fl/fl^ or Cre^–^CXCR4^fl/fl^ females, or from Cre^+^CXCR4^fl/fl^/GFP^+^ male donors to Cre^+^CXCR4^fl/fl^ females as negative control. BM cells were obtained from 6- to 12-week-old C57BL/6J male mice by flushing the BM into cold sterile PBS and filtering the BM through 70 μm mesh. In total, 30 × 10^6^ unfractionated BM cells were injected by retro-orbital i.v. injection into 6- to 10-week-old female recipients on day 0 after conditioning with the BM regimen described above (Figure 6). Following a 3-week recovery, CXCR4 ablation was induced by injecting mice with 75 mg/kg TM for 5 days. For time course experiments to characterize CXCR4 function in pregnancy, female mice were bred with C57BL/6J male mice. The morning of positive vaginal plug was considered E0.5. Successfully bred female mice were killed at various gestational time points between E13.5 and E18.5.

### IHC and immunofluorescence.

Uterine and placental tissues were fixed in 4% paraformaldehyde and embedded in paraffin. Tissue sections (5 μm) were mounted on slides, followed by deparaffinization and rehydration. Slides were then boiled in sodium citrate for antigen retrieval. Sections were incubated overnight with indicated primary antibodies at 4°C (see Supplemental Table 1 for a list of antibodies). The next day, slides were washed 3 times in PBS for 5 minutes and incubated with biotinylated secondary antibody (Supplemental Table 1) for 1 hour at room temperature. For IHC, detection was performed using ABC Vector stain Elite reagents with DAB plus H2O2 (Vector Laboratories). Tissue sections were counterstained with hematoxylin (Sigma-Aldrich). Images of stained sections were obtained using an Olympus BX-51 microscope (Olympus). For immunofluorescence and colocalization studies, DBA (1:50, Vector) and antibodies for GFP and Gzmb were used (Supplemental Table 1), as appropriate, followed by mounting under coverslips using Vectashield fluorescent mounting media with DAPI (Vector Laboratories). Images were captured using laser scanning confocal microscope (LSM 710, Carl Zeiss) and analyzed using ZEN software (Carl Zeiss). Immunoreactions with amplification but without primary and/or secondary antibodies were used as negative controls.

### PAS staining.

PAS staining was done using the standard procedure. Tissue sections were deparaffinized and hydrated with deionized water, before being immersed in PAS stain for 5 minutes at room temperature. Slides were rinsed in several changes of distilled water. Slides were immersed in Schiff’s reagent for 15 minutes at room temperature, followed by washing in running tap water for 5 minutes. Counter-staining of slides was performed in Hematoxylin solution for 90 seconds. Slides were rinsed in running tap water, dehydrated, cleared, and mounted on sections in xylene based mounting media.

### Image quantification and analysis.

For quantitation of proliferating GFP^+^, Gzmb^+^, and DBA^+^ cells in the decidual or placental compartments, 16 high-power confocal microscopy fields (4 high-power fields [HPFs] from each of 4 uterine sections per animal) were assessed for each gestational time point. The total number of DAPI^+^ cells nuclei and DBA^+^ cells was counted in each HPF. For Ly6G staining, the number of Ly6G^+^ cells was counted out of the total cells per tissue section. For quantification of NK cells, granulocytes, and PAS^+^ cells in decidua, ImageJ analysis software (NIH) was used.

### Placental morphometric analysis.

Placental morphometric analyses were performed as described previously (78, 79). ImageJ analysis software was used. All measurements were made on multiple sections taken from a plane in the center of the placenta and perpendicular to its flat side. Morphometric analysis for the estimation of the volume fraction of the decidua, junctional zone, and labyrinth — or that of 3 labyrinthine parameters including fetal vessels, maternal vessels, and trophoblast — were measured by point counting, and results are expressed as percentages of the total number of points. Measurements were performed on 3–5 placentas in each group.

### RNA extraction and qPCR.

Total RNA isolation from decidua and placenta was performed as described previously (40). Briefly, total RNA was extracted from mouse decidual and placental tissues by homogenizing the tissues in 1 mL TRIzol (Invitrogen), followed by the addition of 200 μL chloroform to the lysate for phase separation by centrifugation at 17,115*g* for 15 minutes. RNA in the aqueous phase was then precipitated by addition of 500 μL isopropanol followed by centrifugation at 17,115*g* for 10 minutes. RNA pellet was then washed twice with 1 mL 75% ethanol by centrifugation at 10,354*g* for 5 minutes. The RNA pellet was allowed to dry and then redissolved in nuclease free water. Total RNA was purified via RNeasy spin columns (QIAGEN) followed by treatment with DNase using the TURBO DNA-free kit (Invitrogen). For extraction of total RNA from peripheral blood, total RNA was extracted by disrupting the cells with 300 μL RLT buffer with added β-mercaptoethanol followed by processing in RNeasy spin columns (QIAGEN) as per the manufacturer’s protocol. This was followed by treatment with DNase using the TURBO DNA-free kit (Invitrogen). For each RNA sample, 100–300 ng RNA was reverse transcribed using an iScript cDNA Synthesis Kit (Bio-Rad). qPCR was performed using iQ SYBR Green Supermix (Bio-Rad) on a Bio-Rad CFX96 thermocycler using the gene-specific primer pairs listed in Supplemental Table 2. Gene expression was analyzed on duplicate samples, and the Ct values were normalized to a GAPDH housekeeping gene. We examined Gapdh Ct values between the RT-PCR experimental samples from the different conditions and found that values were similar, with insignificant variation (Supplemental Figure 5). The reaction products were confirmed by 1.5% agarose gel electrophoresis and visualized by UV light after staining with ethidium bromide.

### Tissue processing for scRNA-Seq, single-cell capture, library preparation, and sequencing.

We applied scRNA-Seq using droplet microfluidics (10× Chromium) on a single-cell suspension dissociated from E10.5 uterine implantation site after removal of embryo/placental parts as described above. The uterine tissues were finely minced and subsequently digested with a solution of HBSS (Invitrogen) containing HEPES (25 mM), collagenase B (1 mg/mL; Roche Diagnostics), and deoxyribonuclease I (0.1 mg/mL; Sigma-Aldrich) for 30 minutes at 37°C. Afterward, the cell suspension was filtered sequentially through a 70 μm filter and then through a 40 μm filter, and it was centrifuged at 805*g* for 8 minutes at 4°C. The cell pellet was washed with PBS by centrifugation at 805*g* for 5 minutes at 4°C and then resuspended in PBS with 0.1% FBS. Cell viability (>70% cells alive) and concentration were assessed by the Countess II Cell Counter (Invitrogen) to ensure the quality of cells. Nano-sized droplets that each contain a single cell with the bar-coded gel bead (gel bead in emulsion [GEM]) were generated using the Chromium controller (10x Genomics). The libraries were then created with Single Cell 3′ Library kit V2 according to the manufacturer’s protocol. Reverse transcription was performed with polyT primers containing cell-specific bar codes, unique molecular identifiers (UMIs), and adaptor sequences. All 10x libraries were sequenced in an Illumina HISeq 4000 instrument. We used the 10x Genomics Cell Ranger software v2.0.0 to align to the mm10 and its corresponding gene annotation, to deduplicate, to collect filter bar codes, and to quantify genes.

### scRNA-Seq data processing and UMAP visualization.

scRNA-Seq data from mouse E10.5 implantation site samples were processed using Seurat (v4.3.0.1). The preprocessing consisted of removing cells of low quality with a n_Feature RNA less than 200 and more than 7,500. Cells with mitochondrial reads larger than 10% were also removed. Doublet detection using scDblFinder (v1.12.0) was run to identify populations of doublets, which were then removed in subsequent analysis. The remaining cells were normalized using the NormalizeData function, and the top 2,000 highly variable features were selected using the FindVariableFeatures function in Seurat. The data were then scaled using ScaleData, principal components analysis was performed using RunPCA, and 15 dimensions were used for subsequent analysis. The neighborhoods of cells were found using FindNeighbors, and clustering was performed using FindClusters and a resolution of 0.8. The UMAP embedding of the data was calculated using the RunUMAP function. Canonical marker genes were used to label the clusters to their corresponding cell types. Finally, FindMarkers was used to find differentially expressed genes between NK cells and all the other cells using the Wilcoxon test.

### Statistics.

Statistical information, including *n*, mean, and statistical significance values, is indicated in the text or the figure legends. Statistical analysis was performed using Student’s *t* test (2-tailed), Mann Whitney *U* test, or a 1-way ANOVA with multiple comparisons as appropriate using Prism 9.0 (GraphPad Software). *P* < 0.05 was considered to be statistically significant.

### Study approval.

All animal procedures were performed according to an approved Yale University IACUC protocol (no. 07113).

### Data availability.

The data underlying this article are available in the article, the Supporting Data Values file, and in the supplemental material. The scRNA-Seq data are publicly available in GEO database (GSE241958).

## Author contributions

RT conceived and designed the research study. FL conducted all the major experiments, analyzed the data, and prepared the manuscript draft. FL, YYF, RT, AT, AYC, CB, and JK contributed to experiments, data acquisition, and data analysis. KA and YK contributed to bioinformatic analyses of scRNA-Seq data sets. RT supervised the study and contributed to data interpretation. RT and HST contributed to manuscript revisions. All authors approved the final version and submission of this article.

## Supplementary Material

Supplemental data

Supporting data values

## Version 1 10/10/2023

In-Press Preview

## Version 2 11/08/2023

Electronic publication
