# Supplementary material for: Maternal CXCR4 deletion results in placental defects and pregnancy loss mediated by immune dysregulation
Source: JCI Insight. 2023 Nov 8;8(21):e172216. doi: 10.1172/jci.insight.172216 (PMC10721256; doi:10.1172/jci.insight.172216)
Supplement: Supplemental data [file jciinsight-8-172216-s283.pdf]

Supplementary Figures

Supplementary Figure 1. (A) Litter size of PGRCre+/$CXCR4^{f/null}$ and PGRCre-/$CXCR4^{f/wt}$. (B) Mean litter weight (grams) of PGRCre+/ $CXCR4^{f/null}$ and PGRCre-/$CXCR4^{f/wt}$. N=14-18/group. (C) CXCR4 relative mRNA expression in uterine tissue of PGRCre+/$CXCR4^{f/null}$ and PGRCre-/$CXCR4^{f/wt}$ mice. ***p<0.001.

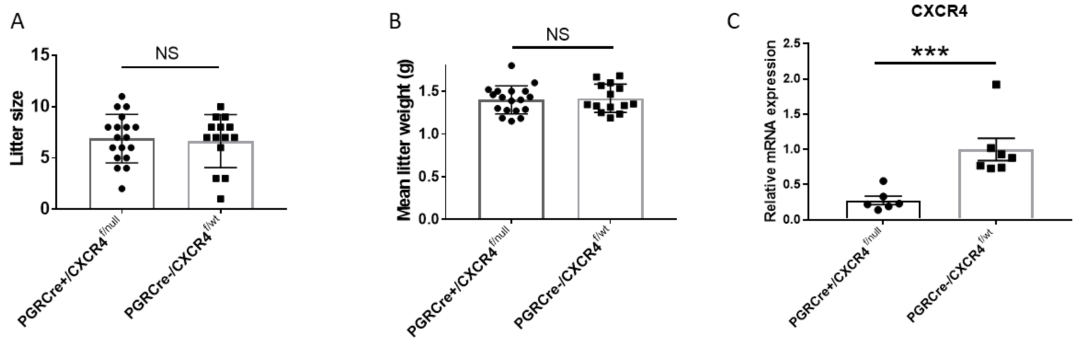

Supplementary Figure 2. (A) Histological sections of decidua from E10.5 WT and CXCR4 KO mice showing immunostaining for the granulocyte marker Ly6G. Red arrows are pointing to more abundant Ly6G+ granulocytes in CXCR4 KO decidua. Bottom image is no primary antibody (PBS) negative control. Scale bar, 50 µm. (B) Quantitation of the number of Ly6G+ cells (granulocytes) per section in decidua of WT and KO mice. **p<0.01.

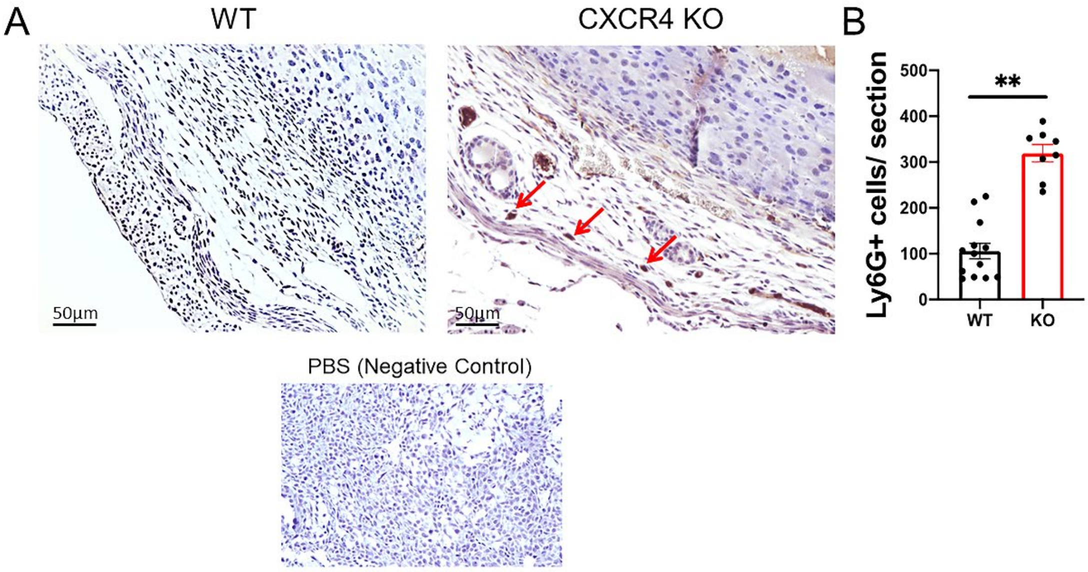

Supplementary Figure 3. (A-B) Immunofluorescence sections of decidua from WT^{WT-BMT}, KO^{WT-BMT} and KO^{KO-BMT} mice. Co-immunostaining showing BM-derived GFP (green) cells, Gzmb (red), DBA+ NK cells (magenta) and DAPI nuclear staining (blue). Upper panel (A) scale bar, 100 µm. Bottom panel scale bar, 50 µm. Note the presence of BM-derived GFP+ cells that are positive for DBA (NK cell marker) and GZMB in KO^{WT-BMT} indicating restoration of functional NK cells with GZMB+ secretory granules, similar to control WT^{WT-BMT}. This is in contrast to KO^{KO-BMT} mice in which all the decidual BM-derived GFP+ cells are devoid of GZMB.

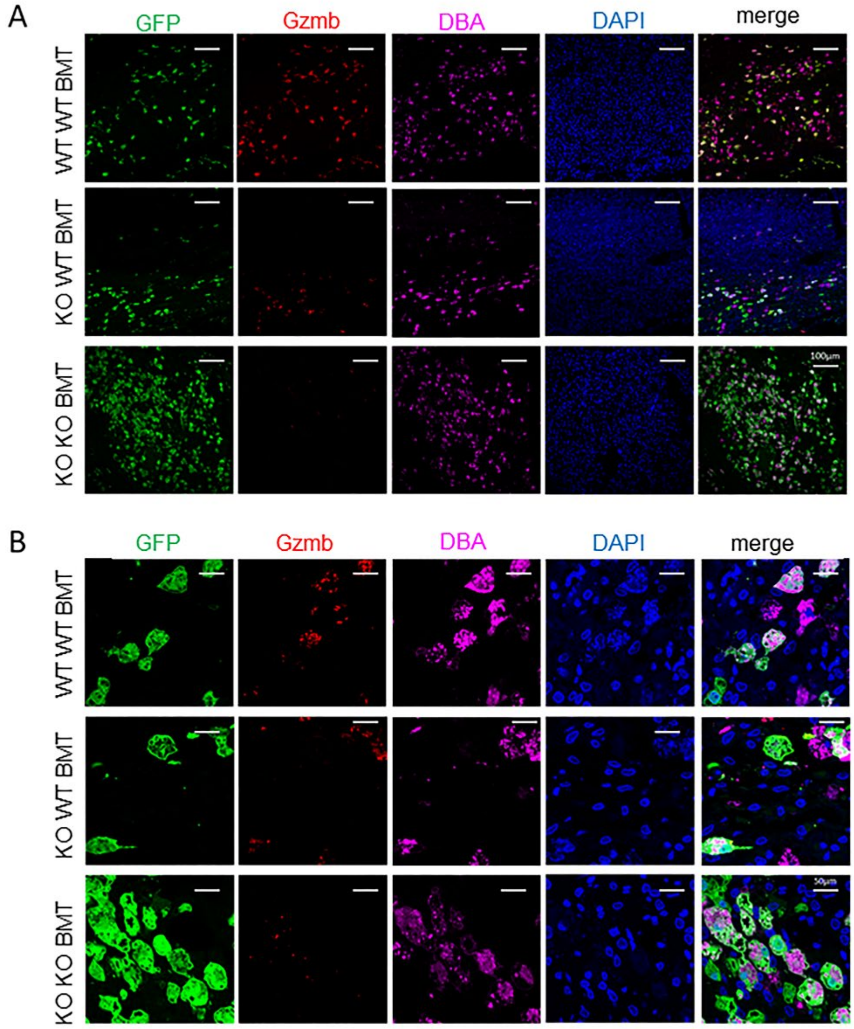

Supplementary Figure 4. Analysis on of a total of 12,738 cells from E10.5 implantation sites of wild-type mice (n=2 mice) by single cell RNA sequencing (scRNAseq). (A) Clustering of cell types following UMAP-based visualization of expression differences for cells using established lineage markers. (B) Expression data dot plots of known lineage markers of all major cell types. Feature plots (C) and (D) expression dot plots of Klrd1, Klre1, Klrg1, Gzmb, Gzmd, Gzme, Gzmf, Gzmg, and Prf1 genes. DCs, dendritic cells; DSC, decidual stromal cell; ECs, endothelial cells; FB, fibroblasts; LECs, lymphatic endothelial cells; Macro, macrophages; Mono, monocytes; NK, natural killer cell; RNA-seq, RNA sequencing; UMAP, Uniform Manifold Approximation and Projection.

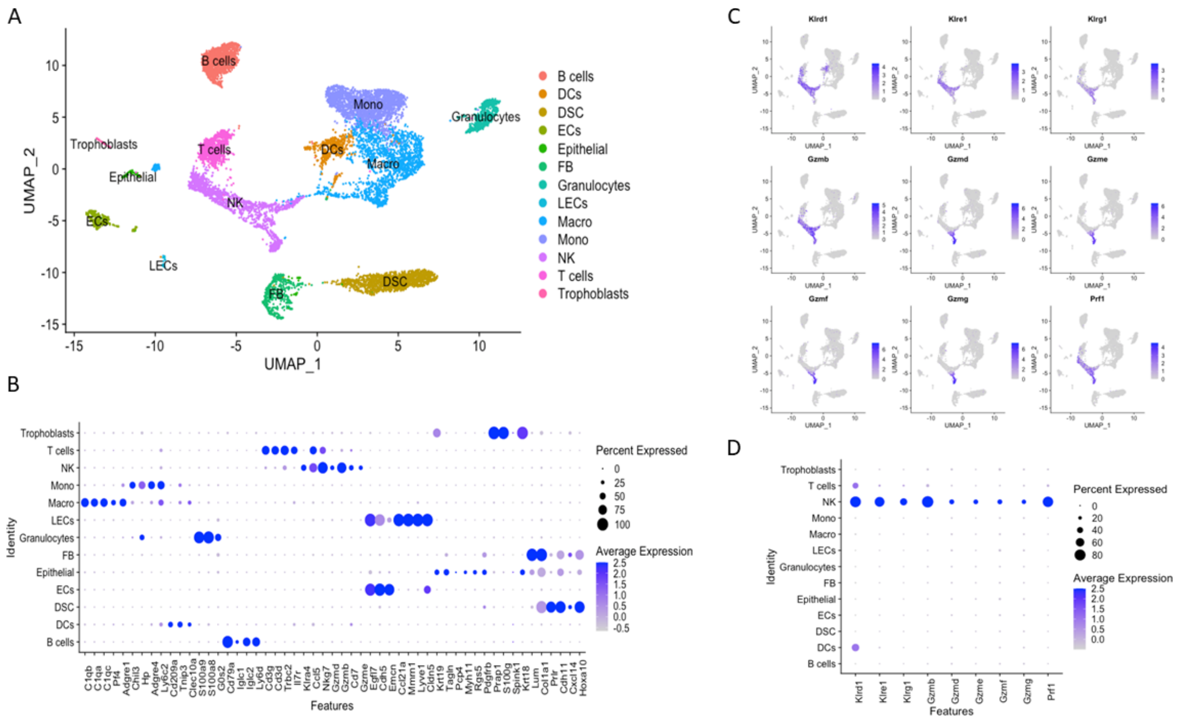

Supplementary Figure 5. Mean GAPDH Ct values of RT-PCR experiments from decidual and placental tissues.

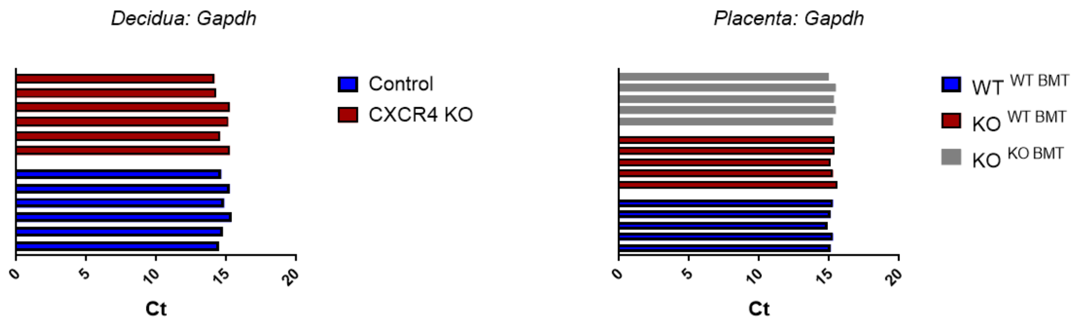

Table 1. Antibodies used.

Antibodies	Source	Identifier
Anti-mouse CD3 Pacific Blue	Biolegend	Cat#100214
Anti-mouse CD25 PE-Cy7	Biolegend	Cat #101904
Anti-mouse CD4 Alexa 700	Biolegend	Cat #108910
Anti-mouse NK1.1 APC-A	Biolegend	Cat #108710
Anti-mouse F4/80 BV711-A	Biolegend	Cat #123147
Anti-mouse Ly6G PerCP	Biolegend	Cat #127654
Anti-mouse CD45 Alexa 605	Biolegend	Cat #103140
Anti-mouse CD11b APC-Cy7	Biolegend	Cat #101226
Anti-mouse Gzmb	Abacm	Cat#ab255598
Anti-mouse CD3	Abacm	Cat#ab135372
Anti-mouse Ly6G	Abacm	Cat#ab25377
GFP	Invitrogen	Cat#G10362
Goat anti-rabbit biotinylated	Vector	BA-1000
Donkey anti-rabbit Alexa Fluor 568	ThermoFisher	A10042

Table 2. Primers used

Gene	Primers	Product (bp)
Ccl2	AGGTGTCCCAAAGAAGCTGTA	79
	GGACCCATTCCTTCTTG	
Ccl3	AGCTGACACCCCGACTGCCT	116
	TCAGGAAAATGACACCTGGCTGGGA	
Ccl5	AGCCAGCTGTGGTATTCCTGACCA	81
	TCATGTACTCAGTGACCCAGGGCT	
Ccr5	ATTCTCCACACCCTGTTTCG	388
	GTTCTCCTGTGGATCGGGTA	
Cxcr4	AGAAGCTAAGGAGCATGACGG	223
	GCGTGGACAATAGCGAGGT	
Cxcl12	GAGCCAACGTCAAGCATCTG	101
	CGGGTCAATGCACACTTGTC	
Il1b	GGACCCCAAAAGATGAAGGGCTGC	99
	GCTCTTGTTGATGTGCTGCTGCG	
Il6	CCTCTCTGCAAGAGACTTCC	87
	CTCCGGACTTGTGAAGTAGG	
Il12b	AACCAGAAAGGTGCGTTCCTC	118
	ATGCCCACTTGCTGCATGA	
Cxcl1	CCTGCAGACCATGGCTGGGAT	98
	GTGTGGCTATGACTTCGGTTTGGG	
Cxcl2	GTTTGCCTTGACCCTGAAGCCCC	80
	CCAGGTCAGTTAGCCTTGCCTTTGT	
Csf2	TCGAGCAGGGTCTACGGGGC	101
	GTCCGTTTCCGGAGTTGGGGG	
Il8	*TTCAGAGACAGCAGAGCACA*	170
	AGCACTCCTTGGCAAAACTG	
Vegfa	GTGCACTGGACCCTGGCTTTA	147
	GGTCTCAATCGGACGGCAGTA	
Angpt2	GCTGAAGGACTGGGAAGGC	70
	GGACTCTTCACCAGCGAGGTA	
Hif1α	*GCAGCAGGAATTGGAACATT*	150
	GCATGCTAAATCGGAGGGTA	
Tnfa	ATGGCCCAGACCCTCACACTCA	80
	TGGTGGTTTGCTACGACGTGGG	
Tgfβ1	*GTTGAGGAAACAAGCCCAGA*	199
	GTTACCCAGGCTGGTCTCAA	
Nfκb	TCCACTGTCTGCCTCTCTCGTC	197
	GCCTTCAATAGGTCCTTCCTGC	
Lox-1	GTGGTTCCCTGCTGCTATGAC	127
	CTGAGTAAGGTTCGCTTGGTATTG	
Il10	GCGGCTGAGGCGCTGTCAT	109
	GGCCTTGTAGACACCTTGGTCTTGG	

Il18	GAGGGCCACACAAGTCGC	157
	GCTGGGCCAGAATGATGTGA	
Klrd1	GGCCTCTGTGGTGGACTAAC	243
	TGTTCCTTCTGAGTGCCGAC	
Klre1	TAAGAGACAAGCAGGCACGCTGACTG	433
	ATGGATGAAGCACCTGTAACCCG	
Klrg1	GGCTTGAGGAACATTGATGG	76
	TCAAGCTGTTGGTAAGAATCCTC	
Prf1	ATGAGCAGAGCCCGGTGGCA	127
	GTCGTGGGCAGCAGTCCTGG	
Gzmd	AGCTGGAGCAGAGGAGATCA	172
	TTGGACAGAGCTGTTTTTGC	
Gzmg	GATTCTCCTGACCCTACTTC	547
	CTGCGTGGTCTTGGAATAGG	
Gapdh	GCCTGCTTCACCTTCTT	188
	ATGGCCTTCCGTGTTCCTAC	
Gzmb	GATATGTGGGGGCTTCCTTA	382
	CTCACACTCCCGATCCTTCTG	
Gzme	ACCTCCTTCCTCCCCTTCC	130
	CTCCTCTGCTCCAGCTCCA	
Gzmf	CACTGGAAGCTCAATGAGAGTCATAC	134
	TGATGTCACTGGTGTTGTCCTTATC	
Ifng	CTTCTTCAGCAACAGCAAGG	101
	TGAGCTCATTGAATGCTTGG	
Ctsw	CACTCTGTCTTGCTGGTGGG	103
	TCCAGTATGGGGAGGAGTGG	
Igf2	CGCTTCAGTTTGTCTGTTCG	95
	GCAGCACTCTTCCACGATG	
Lifr	CTTCGATCCTCAACACAGAGC	360
	TGGTTAGTGCACCCATAGAGG	
Plgf	TGCTGGTCATGAAGCTGTTC	222
	GGACACAGGACGGACTGAAT	
Tpbpa	AAATGAGTGCCTCCGGTCAG	152
	AAACCCATCGCCACTCTCTG	
Tgfb1	TATACTGAGACACCTTGG	83
	GTGATAGTCCTGAATAATTTG	
